# Current Insight into Human Ornithine Aminotransferase: A Review

**DOI:** 10.1002/prot.70134

**Published:** 2026-03-26

**Authors:** Fulvio Floriani, Carla Borri Voltattorni, Riccardo Montioli

**Affiliations:** ^1^ Department of Neuroscience, Biomedicine, and Movement Sciences, Biological Chemistry Section University of Verona Verona Italy

**Keywords:** molecular effects of mutations, ornithine aminotransferase, pathogenic variants, pyridoxal‐5′‐phosphate

## Abstract

Human ornithine aminotransferase (hOAT) is a mitochondrial matrix pyridoxal‐5′‐phosphate enzyme (PLP) that catalyzes the reversible transfer of the δ‐amino group of L‐ornithine (L‐Orn) to α‐ketoglutarate (α‐KG) yielding glutamate‐5‐semialdehyde (GSA) and glutamate. GSA is prone to cyclize to Δ1‐pyrroline‐5‐carboxylate. Human OAT holds significant clinical and scientific interest because (i) its dysfunction causes gyrate atrophy (GA) of the choroid and retina, a rare autosomal recessive disease, and (ii) it is recognized as a potential target for chemotherapeutic drug development, being overexpressed in some types of cancer. Here, we review the kinetic and structural features of the enzyme, as well as the mechanistic aspects of hOAT inhibition. Moreover, we focus our attention on the characterization of the structural and functional properties of the artificial variants and of those associated with GA. Considering that great progress toward the characterization of the pathogenic variants has been reached in the last few years, we summarize here, by revisiting the data available on the hOAT and its variants as purified recombinant form, the current understanding of (i) the molecular defect(s) of studied disease‐causing mutations and (ii) the residues (particularly, active site residues critical for dictating the reaction specificity) and/or regions of the enzyme crucial for its folding and/or catalytic properties.

**Abbreviations**
GSA: glutamyl‐5‐semi‐aldehyde
L‐Orn: L‐ornithine
OAT: ornithine aminotransferase
P5C: Δ1‐pyrroline‐5‐carboxylate
PLP: pyridoxal 5′‐phosphate
PMP: pyridoxamine 5′‐phosphate
wt: wild type
α‐KG: α‐ketoglutarate

## 1 Introduction

Ornithine δ‐aminotransferase (OAT) (E.C. 2.6.1.13) is an enzyme found in almost all eukaryotic organisms, from protozoa to higher animals and plants.

**FIGURE 1:**
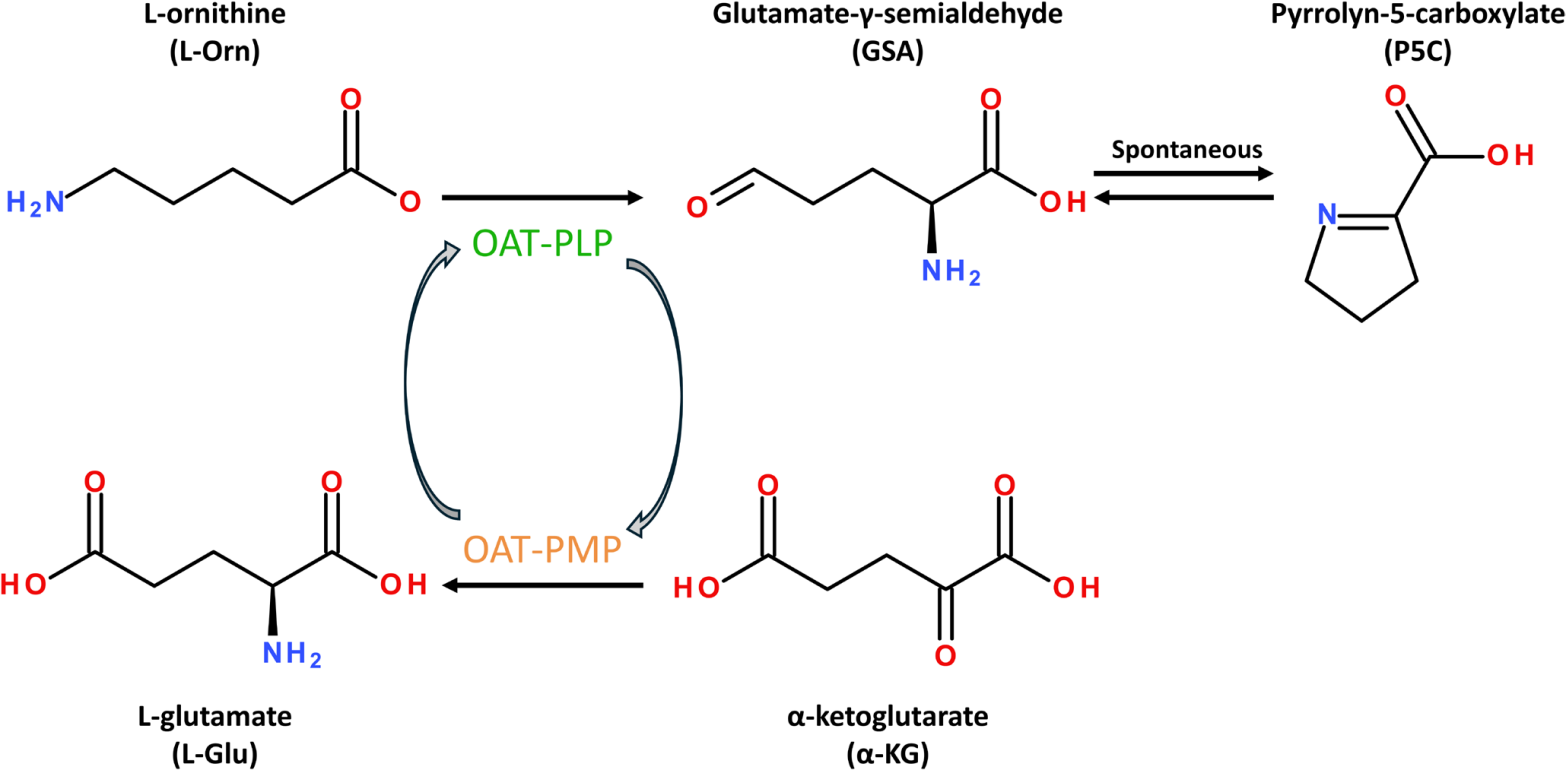
. Catalytic cycle of hOAT. In the first half‐reaction, L‐ornithine is converted into GSA with the concomitant conversion of the coenzyme PLP into PMP. The product GSA spontaneously cyclizes, forming P5C. In the second half‐reaction, α‐ketoglutarate is converted into glutamate with the regeneration of the PLP form of the coenzyme.

In all these species, OAT is a soluble, intracellular protein. In fungi and in most cellular organisms, OAT is a cytosolic enzyme, while it localizes in the mitochondrial matrix in most plants and vertebrates [1]. In mammals' organs, OAT is widely expressed with the highest concentrations in liver, intestine, and kidneys [2]. Like all amino‐transferase enzymes, OAT requires pyridoxal‐5′‐phosphate (PLP) to be functional. Indeed, the enzyme catalyzes the conversion of L‐ornithine (L‐Orn) into glutamyl‐5‐semi‐aldehyde (GSA) and vice versa, using α‐ketoglutarate (α‐KG) and L‐glutamate (Glu) as co‐substrates by following a classical ping‐pong mechanism (Figure 1). In the first half‐reaction, OAT with the co‐enzyme in the PLP form reacts with L‐Orn, generating GSA and the enzyme in the pyridoxamine 5′‐phosphate (PMP) form. Then, in the second half‐reaction, OAT in the PMP form binds α‐KG and converts it to Glu, thereby regenerating OAT in the PLP form (Figure 1). Hence, the enzyme, belonging to subgroup II of the extensively investigated aminotransferase family [3, 4], is an ω‐aminotransferase in that it catalyzes the transamination of the distal amino group of Orn in the first half‐transamination, while it catalyzes an α‐transamination in the second transamination reaction. The GSA undergoes spontaneous cyclization through the reaction between the amine and aldehyde functions, forming Δ^1^‐pyrroline‐5‐carboxylate (P5C), a compound that is in chemical equilibrium with GSA. Nuclear magnetic resonance studies, performed using deuterated water, indicated a pH dependence of the GSA/P5C equilibrium where, above neutral pH, the cyclic form resulted predominant [5]. This suggests that in the mitochondrial matrix, where pH is reported around 7.9–8.0 [6], the equilibrium is shifted toward the P5C formation. However, it must also be considered that since the reaction reaches equilibrium quickly, even a minimal amount of GSA can be used by OAT for the reverse reaction. Thus, depending on the equilibrium between GSA and P5C, a double role can be envisaged for OAT. The enzyme can be related to Glu metabolism (bioenergetics, nitrogen flux, acid–base metabolism) but also to proline and its related metabolic pathways.

## 2 Human OAT

A great part of the information regarding OAT derives from studies focused on human OAT (hOAT). The enzyme is codified by the nuclear gene *OAT* (MIM#613349) on chromosome 10q26.13 [7], synthesized as a 48 385 kDa precursor in the cytosol, and imported into mitochondria where it reaches the functional conformation upon the removal of a N‐terminal mitochondrial targeting sequence (residues 1–25), thereby generating a ~45 kDa mature protein [8]. The overall transamination of the pair Orn/α‐KG catalyzed by hOAT in the purified recombinant form is characterized by a *k*_cat_ value of ~35 s^−1^, and K_m_ values of about 6.5 and 3.9 mM for L‐Orn and αKG, respectively [9, 10]. Kinetic and spectroscopic experiments aimed to unravel the relative specificity of hOAT for amino donor and amino acceptor substrates, indicating that (i) the L‐Orn analogues, L‐2,4‐diamino butyric acid, 5‐aminovaleric acid and ɤ‐aminobutyric acid exhibit relatively slow catalytic rates in comparison with L‐Orn [11], and that (ii) the α‐KG analogues, oxaloacetate, α‐ketobutyrate, and pyruvate display *k*_cat_ lower and K_m_ values significantly higher than those of α‐KG [10]. Stopped‐flow experiments provided evidence for the pH dependence of the half‐reactions of hOAT. Similar pK values were determined for the initial and rate‐limiting step of both the first and second half‐reactions, and the protonation state of the PLP‐binding Lys392 has been suggested to be responsible for these dependences [11]. The hOAT‐bound coenzyme gives rise to two absorbance bands, at 420 and 340 nm, associated with positive dichroic signals at the same wavelengths. Upon excitation at 420 or 340 nm, fluorescence emission spectra of holo‐hOAT display a maximum around 505–510 nm. Overall, these data allowed to attribute the 420 and 340 nm absorbance and CD bands to the ketoenamine and enoliminic tautomeric forms of the internal aldimine [9]. Furthermore, although the PLP binding to apo‐hOAT does not affect the dichroic signal in the near‐UV region, it causes a pronounced reduction of hydrophobic surfaces of the molecule and a consistent increase of the melting temperature value. These data clearly suggest that the apo and the holo forms of hOAT are characterized by different conformations [9].

**FIGURE 2:**
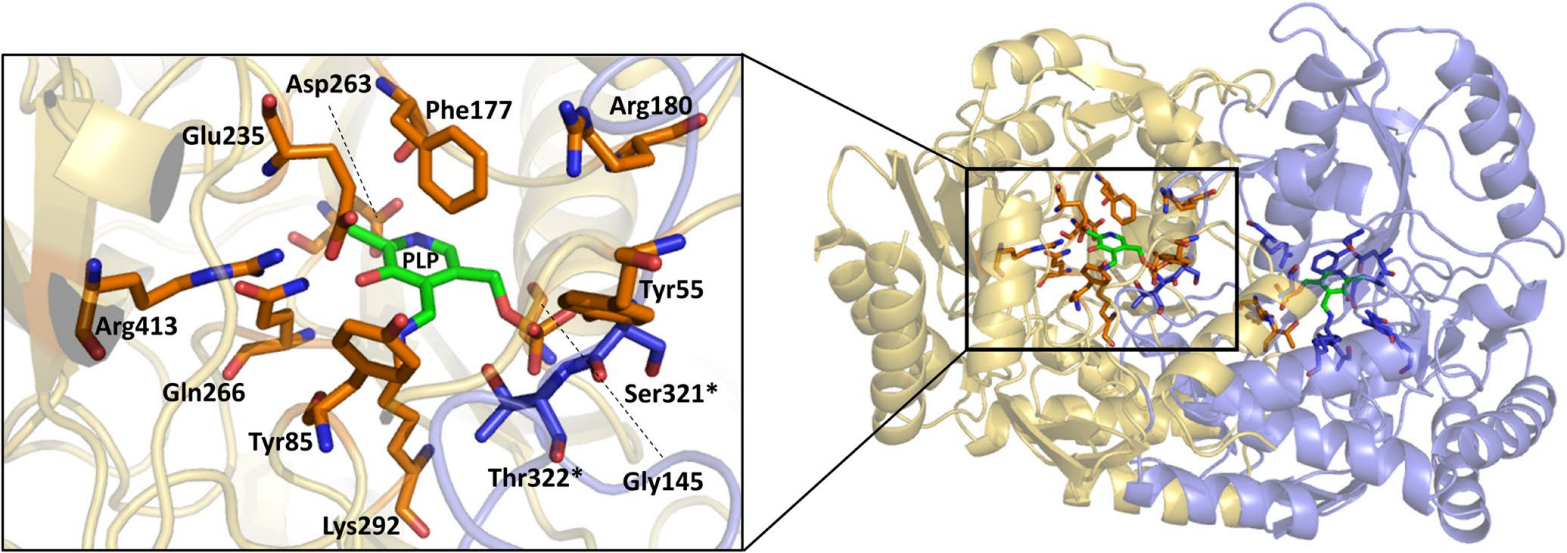
. Active site architecture of human OAT. Cartoon representation of the dimeric structure of human OAT where monomers are colored yellow and blue, respectively. In the magnified detail, the residues involved in the PLP binding or in the interaction with the substrates are indicated and represented as sticks. *Denotes residues belonging to the blue neighboring subunit. Image was rendered by PyMol (Schrödinger).

*hOAT structure*‐The crystal structure of the holo PLP form of hOAT was solved at 2.5 Å [12]. The overall conformation of the enzyme consists in a hexameric assembly composed of three homodimers held together mainly by electrostatic interactions. Each monomer of the dimeric unit contains 12 α‐helices and 14 β‐strands, and includes (i) a N‐terminal segment (residues 25–95), which partly overlaps the large domain of the adjacent subunit, (ii) a large domain (residues 96–345) comprising the active site region and most of the subunits interface, and (iii) a C‐terminal small domain (residues 346–439). In the homodimer, the two active sites are located at the subunit interface and are composed by residues belonging to both the monomers. Each active site hosts a PLP molecule which is covalently bound by a Schiff base linkage with Lys292 and interacts with residues belonging to both subunits (Asp263, Phe177, Gln266, Gly142, Ser321*, Thr322*). The substrate binding site is located in front of the PLP‐Lys292 internal aldimine, and is delimited by the side‐chains of Tyr55 and Tyr85 and by three charged residues Arg180, Glu235 and Arg413 (Figure 2). Moreover, in the free enzyme, Arg413 and Glu235 side chains were found to be involved together in a salt bridge. This couple of residues are highly conserved among all transaminases [13]. In the external aldimine formed with L‐Orn, the α‐amino and α‐carboxylate groups are engaged by the side chain of Tyr55 and Arg180, respectively [14]. The Arg413 side chain was also found to be important to interact with the γ‐carboxylate group of L‐Glu and it is supposed to compete with Arg180 for the binding to the α‐carboxylate group of L‐Orn in the first half reaction as it was observed in other aminotransferases [15]. For this reason, Glu235 was suggested to play the role of shielding Arg413 by a salt bridge in the L‐Orn external aldimine formation by a mechanism called “Glu235‐switch” that implies the change of the Glu253 position between the first and the second half reaction [14]. The mobility of the Glu253 side chain was supported also by the analysis of the crystal structure of hOAT as a function of pH, revealing the absence of the Arg413‐Glu235 salt bridge at pH 6.0 [11].

About the quaternary structure, although the crystallographic conditions seemed to favor a hexameric structure [12, 16], ultracentrifugation and size exclusion chromatographic experiments revealed that both holo and apo hOAT exhibit a tetrameric structure, even if the hOAT holo form displays a tetramer‐dimer equilibrium dissociation constant 5‐fold lower than the apo form does [9]. Besides, it is noteworthy that the apo form of hOAT is thermally unstable and prone to unfolding and aggregating in physiological conditions. This indicates that the PLP binding promotes the shift of the equilibrium of dissociation toward the tetrameric assembly and increases the thermal stability of the enzyme [9]. However, the possible biological role of the tetrameric assembly of OAT remains an open question.

*hOAT inhibitors—Wide* is the medical interest of hOAT. Indeed, high expression levels of the enzyme have been observed in some types of cancer. In particular, it was found that the enzyme is overexpressed in hepatocellular carcinoma and the inactivation of OAT inhibits the growth of such type of cancer [17]. Moreover, it was found that the knockdown of hOAT suppresses tumor growth in a mouse model of non‐small cell lung cancer (NSCLC) [18]. The identification by Wang et al. [19] of hOAT as a diazonamide‐binding protein allowed to unravel a paradoxical and unexpected function for OAT in mitotic cell division, therefore identifying the enzyme as a target for chemotherapeutic drug development.

These data explain the great interest focused on the search for highly potent and selective hOAT inhibitors. The identified hOAT inhibitors were L‐canaline ((S)‐2‐amino‐4‐amino‐oxybutyric acid), a natural compound analogue of ornithine [20], and L‐fluoromethylornithine (5‐FMO) [21]. Both are irreversible mechanism‐based inhibitors, but while L‐canaline was found to inhibit other PLP enzymes [22], 5‐FMO resulted to be highly specific for hOAT [21]. The crystal structure of hOAT complexed with 5‐FMO provides evidence for an inactivation mechanism based on the formation of an enamine intermediate, as previously suggested by Bolkenius et al. [23]. More recently, based on the catalytic similarities existing between hOAT and GABA aminotransferase, several mechanism‐based inhibitors have been developed, and some of them are particularly selective for hOAT over GABA‐AT [24, 25, 26, 27, 28]. Moreover, OAT inhibition was suggested to be promising also as a therapeutic approach for preventing neurotoxicity and mortality in acute hyperammonemia [29].

## 3 Characterized hOAT Variants

**FIGURE 3:**
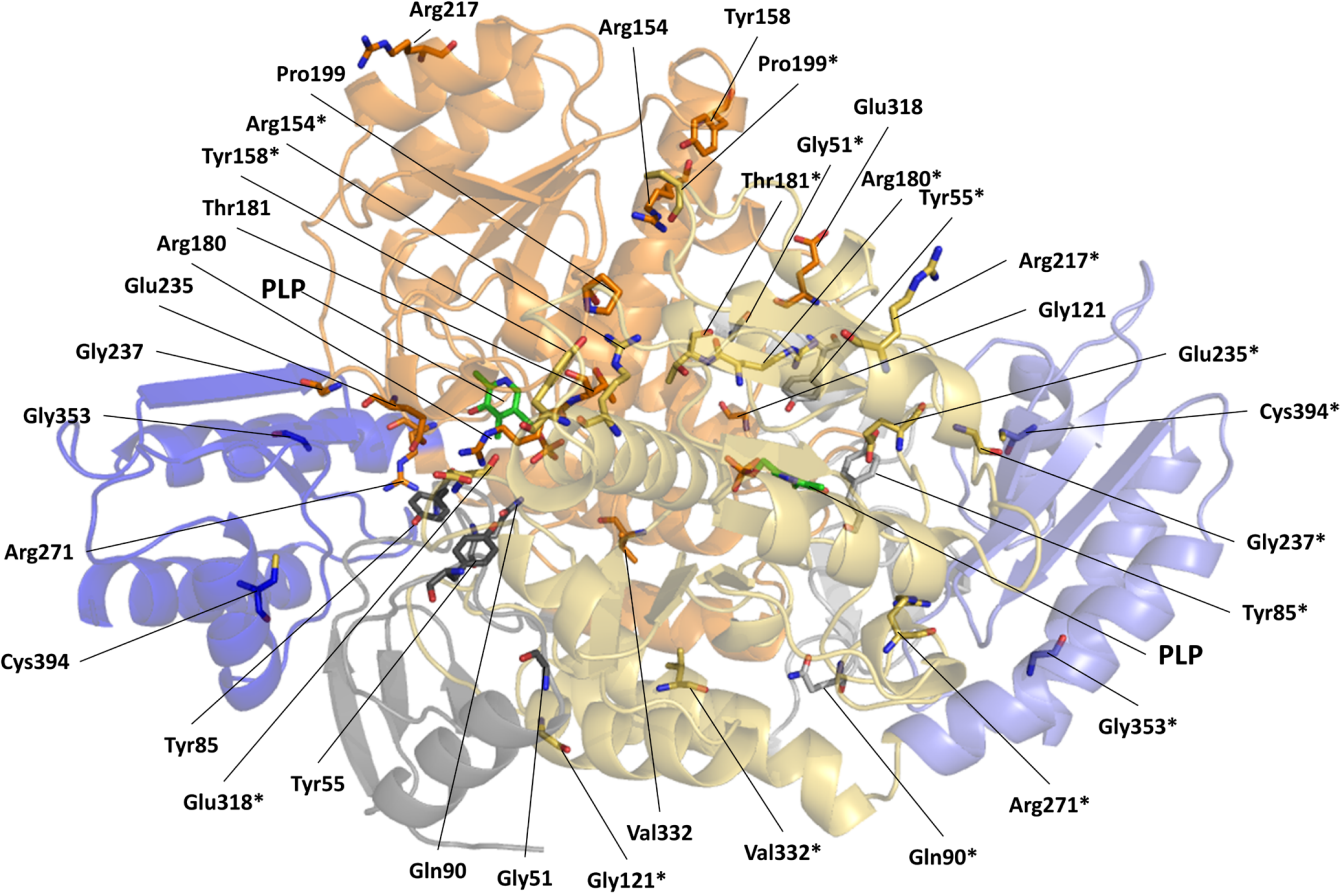
. Structural map of the characterized mutation sites. Cartoon representation of the dimeric structure of hOAT. N‐terminal, C‐terminal and large domain are colored in gray, blue and orange, respectively. Monomers are highlighted by dark and light colors. Mutation sites are indicated and represented as sticks. *Denotes residues of the neighboring subunit. Image was rendered by PyMol (Schrödinger).

The malfunction, or deficit of hOAT is responsible for gyrate atrophy of the choroid and retina (GA), an autosomal recessive hereditary disorder leading to a progressive loss of vision [30]. Due to the reduced or absent ornithine transaminase activity, patients affected by GA present elevated L‐Orn levels in plasma and other body fluids. This is suggested to have an important role in the pathogenetic mechanism of the disease [31, 32]. To date, 58 pathogenic point mutations have been identified (Table 1) according to OMIM database (https://www.omim.org) and/or the Human Gene Mutation Database (https://www.hgmd.cf.ac.uk) (both accessed in June 2025). Among them, a group of missense variants, together with artificial variants, have been investigated at a molecular level providing information about the structural and/or catalytic role of the affected residues, and the molecular alterations caused by the amino acid substitutions (highlighted in red in Table 1). An overview of the structural position of such mutations is illustrated in Figure 3. The majority of them (67%) involves residues belonging to the large domain. Among the remaining, four mutations concern residues (Gly51, Tyr55, Tyr85, and Glu90) belonging to the N‐terminal domain, while two mutations affect residues (Gly353 and Cys394) lying in the C‐terminal domain. Two clusters of mutations that map either at the active site or at the subunits interface, deserve to be underlined. Instead, other mutations affect residues located in peripheral regions.

**TABLE 1:** Pathogenic and artificial hOAT variants.

Mutated residues	Protein variants	Domain	Known molecular alterations caused by the amino acid substitution/s	References
Met1	M1L	N	—	[43, 44]
Gly51	G51D	N	Reduced PMP binding affinity; significant residual catalytic activity (about 1/10 of wt K); alteration of the tetramer‐dimer equilibrium	[36, 45]
Asn54	N54K	N	—	[38]
Tyr55	Y55H/Y55A ^a^/Y55G ^a^	N	Catalytic efficiency < 1/100 of wt, reduced substrate affinity (K_m_ for L‐orn 10–20‐fold higher of wt)	[34, 45, 46]
Leu62	L62P	N	—	[47]
Ser83	S83N	N	—	[48]
Tyr85	Y85I	N	Reduced substrate binding affinity (K_m_ value for L‐Orn 100‐fold higher of wt K); reduced catalytic efficiency (≈1/1000 of wt). Altered substrate specificity	
Asn89	N89K	N	—	[46]
Gln90	Q90E		Reduce folding efficiency. Slightly reduced catalytic activity	[39, 49]
Gly91	G91E	N	—	[45]
Cys93	C93F	N	—	[46]
Gln104	Q104R	Large	—	[50]
Gly121	G121D	Large	Reduced PMP binding affinity; significant catalytic activity (initial velocity about 1/10 of wt); alteration of the tetramer‐dimer equilibrium	[36, 45]
Glu125	E125del	Large	—	[51]
Gly142	G142E	Large	—	[51]
Arg154	R154L/R154H	Large	Strong reduction of the catalytic activity (not measurable); reduced thermal stability; alteration of the tetramer‐dimer equilibrium	[36, 39, 46, 52]
Tyr158	Y158S	Large	Catalytic efficiency ≤ 1/10 of wt, mainly due to the decreased k_cat_ value and increased K_m_ for α‐ketoglutarate. Reduced thermal stability and alteration of the tetramer‐dimer equilibrium	[36, 53]
Tyr166	Y166term	Large	—	[54]
Trp178	W178term	Large	—	[55]
Arg180	R180T	Large	Strong reduction of the catalytic efficiency (less than 1/1000 of wt) due to both reduced k_cat_ value (1/100 of wt k_cat_) and strong increased value of K_m_ for L‐Orn (40‐fold of wt K_m_). Altered substrate binding orientation	[33, 56]
Thr181	T181M	Large	Reduction of the catalytic efficiency (about 1/70 of wt), due to both the increased K_m_ value for L‐Orn (15‐fold of wt K_m_) and reduced k_cat_ (1/10 of wt k_cat_). Alteration of tetramer‐dimer equilibrium	[36, 51, 57]
Ala184	A184T	Large	—	[47, 58]
Asp195	D195Y	Large	—	[51]
Pro199	P199Q	Large	Strong reduction of the catalytic efficiency (≤ 1/500 of wt) mainly due to the decreased k_cat_ values. Reduced thermal stability and alteration of the tetramer‐dimer equilibrium	[36, 50, 59, 60]
Tyr209	Y209term	Large	—	[59]
Arg217	p.R217A ^a^	Large	Loss of the tetrameric structure	[9, 10]
Ala226	A226V	Large	—	[61]
Gln233	Q233R	Large	—	[62]
Glu235	p.E235A ^a^/S ^a^	Large	Reduced catalytic efficiency of the variant E235A (1/1000 of wt) mainly due to the increase of the K_m_ value for L‐Orn (about 60‐fold of wt). Unlikely, E235S display only about 8‐fold reduction of the catalytic efficiency for L‐Orn	
Gly237	G237D	Large	Strong reduction of the catalytic activity. Kinetic parameters not measurable	[39, 63]
Pro241	P241L	Large	—	[46, 47]
Tyr245	Y245C	Large	—	[46, 56]
Arg250	R250P/R250term	Large	—	[45, 46, 47]
Thr267	T267L	Large	—	[46]
Ala270	A270P	Large	—	[46, 56]
Arg271	R271K	Large	Reduced folding efficiency. Significant residual catalytic efficiency (about 1/2 of wt)	[39, 43, 46]
Trp275	W275term	Large	—	[55]
Gly297	G297C	Large	—	[64]
Arg299	R299term	Large	—	[59, 65]
Glu318	E318K	Large	Reduced folding efficiency. The catalytic efficiency resulted similar to that of wt.	[39, 50, 59, 66]
His319	H319Y	Large	—	[51, 67]
Asn326	N326K	Large	—	[51]
Arg331	R331term	Large	—	[50]
Val332	V332M	Large	Reduced folding efficiency. Reduced PMP binding affinity. Catalytic efficiency about 1/2 of wt.	[9, 38, 50]
Gly353	G353D	C	Reduced folding efficiency. Catalytic activity not evaluated	[40, 46, 47]
Gly373	G373E	C	—	[59]
Gly375	G375A	C	—	[43, 46]
Trp391	W391TERM	C	—	[47]
Cys394	C394Y/C394R	C	Reduced folding efficiency. Significant catalytic efficiency (about 1/2 of wt)	[39, 46, 47, 50]
Arg396	R396TERM	C	—	[46, 56]
Arg398	R398TERM	C	—	[61]
Gly401	G401TERM	C	—	[46, 47]
Leu402	L402P	C	—	[56]
Pro417	P417L	C	—	[46, 50]
Arg426	R426TERM	C	—	[57, 59]
Ile436	I436N	C	—	[50]
Leu437	L437F	C	—	[46]

*Note:* The variants characterized at a molecular level are highlighted in red.

^a^ Artificial variants.

### 3.1 Active Site

Significant contributions to comprehending the role of some residues were provided by the study of the artificial variants produced at the active site level. In particular, Y55A/G, Y85I, and E285A, as well as the pathogenic variant R180T [33, 34], have been deeply analyzed. A comparative study between OAT and GABA‐AT indicated that both Y55 and T85 are major contributors to the productive binding of L‐ornithine. Moreover, the presence of tyrosine or isoleucine at position 85 was found to be crucial for the enzyme to discriminate between L‐Orn and GABA [34]. In addition, the same study gained insight into the “Glu235 switch” hypothesis previously suggested by Storici et al. (1999) [14]. In fact, the study of the artificial E285A/S variants suggested that the major contribution provided by the interaction between Arg413 and Glu235 is to limit the reaction with glutamate more than to favor the binding of L‐Orn [34].

The active site Arg180 residue was first identified as a substrate binding residue by crystallographic analyses [12, 14, 35]. Thereafter, a significant step directed toward the understanding of the role of Arg180 was provided by the kinetic and structural characterization of the pathogenic R180T‐hOAT variant in its purified recombinant form [33]. In detail, despite the absence of significant structural alterations assessed by both spectroscopic and crystallographic analyses (pdb file 6HX7), the R180T variant exhibited a dramatic reduction of the δ‐transamination catalytic efficiency due to both a reduced substrate binding affinity and a reduced *k*_cat_ value. Moreover, the variant showed the ability to catalyze, in addition to the δ‐transamination, the α‐transamination of L‐ornithine, thereby producing pyrroline‐2‐carboxylate (P2C). Based on these findings, it was suggested that Arg180 is not only responsible for the L‐ornithine binding affinity, but also plays a role in the correct orientation of the substrate in the hOAT active site [33].

### 3.2 Subunits Interface

In our labs, Arg217 was identified as an important hot‐spot at the dimer‐dimer interface of hOAT. The characterization of the R217A mutant revealed that this artificial variant has a dimeric structure endowed with spectroscopic and catalytic features, as well as thermal stability comparable to the corresponding ones of the tetrameric form of hOAT [9]. These studies strongly indicate that the dimer is the catalytic unit of hOAT and suggest that the tetrameric structure is not involved in the catalysis or in the stability of the enzyme as well. Then, Pampalone et al. [10] evaluated both the native tetrameric form and the dimeric R217A variant of hOAT loaded in red blood cells as possible tools for enzyme replacement therapy.

The dimeric unit of hOAT is accompanied by a wide surface of interactions occurring between the monomers involving both active sites [12]. The characterization of the six pathogenic variants affecting residues located at the monomer‐monomer interface (Gly51, Gly121, Arg154, Ser158, Thr181 and Pro199) shed light on the effects of the interface perturbations on the hOAT properties [36]. Although to a different extent, all the six substitutions G51D, G121D, R154L, S158F, T181M and P199Q reduced the thermal stability of the enzyme and shifted the tetramer‐dimer equilibrium toward the dimeric form: in fact, all R514L, S158F, T181M and P199Q mutants were found to be completely dimeric, while both G51D and G121D mutants exhibited an increased tetramer‐dimer equilibrium constant value with respect to that of the wild‐type [9]. Nevertheless, unlike the artificial R217A dimer, these variants showing a perturbed monomer‐monomer interface display a strongly compromised catalytic activity. Indeed, the catalytic activity of R154L was undetectable, while that of the S158F, T181M and P199Q exhibited a drastic activity reduction with respect to the one of the wild‐type hOAT. This implies that these residues play a role in the acquisition of the proper active site architecture and of the conformation of the dimer‐dimer interaction surface. This is again consistent with the finding that these mutations alter the PLP microenvironment.

A peculiar catalytic defect was observed for both G51D and G121D variants which resulted in a rapid inactivation in turn ascribable to the dissociation of the PMP form of the coenzyme during the catalysis [36]. Such a phenomenon was reported for another hOAT variant (discussed later) [37] and causes the conversion of the enzyme into the unstable OAT apo‐form.

*Peripheral enzyme regions*. The effects of the amino acid substitutions Q90E, G237D, R271K, E318K, V332M, G353D and C394Y, affecting residues located outside the active site and the interface regions, were investigated. Significant advances in the understanding of the molecular defects of hOAT variants were obtained by the study of the V332M variant [37]. Integrated molecular and cellular analyses of this variant revealed that, beside slight structural and catalytic alterations, V332M‐hOAT is characterized by a defective binding of the pyridoxamine form of the coenzyme. Hence, although the variant was found able to bind PLP and to efficiently catalyze the first half reaction, it resulted prone to release the PMP, and to convert itself into the apoform during the catalysis [37]. Furthermore, when expressed in a cellular model of pathology the V332M variant resulted present as an inactive apo‐dimeric form that partly shifts to the active holo‐tetramer upon binding exogenous PLP. For this reason, the authors suggested that this could explain the responsiveness of patients bearing the V332M mutation to pyridoxine administration [37, 38]. It is noteworthy that the same molecular defect is shared by the G51D and G121D variants mentioned above [36], but little is known about the B6 responsiveness of homozygous patients bearing these mutations. The presence of this molecular defect and its relation with the responsiveness to the vitamin B6 treatment of the patients certainly deserve further investigations among the hOAT variants.

Based on studies focused on a human cellular model of disease, Q90E, R271K, E318K, and C394Y mutations resulted to be folding‐defective variants. This is because, although to a different extent, they displayed reduced protein levels due to increased degradation and/or aggregation propensity [39]. In addition, the analysis on the purified protein revealed that while the Q90E, R271K, and E318K mutations affect mainly the folding efficiency of the enzyme, the C394Y mutation also showed a consistent alteration of the kinetic parameters, together with a substrate inhibition event. Then, the G353D variant exhibited a very low solubility and aggregation propensity, and for these reasons, was identified as a folding‐defective variant [40]. Finally, the purified G237D variant was identified mainly as a catalytic‐defective variant, suggesting a crucial role of the Gly237 residue in the proper acquisition of the catalytic ability of hOAT [36].

## 4 Identification of Structural and/or Functional Residues in hOAT

**FIGURE 4:**
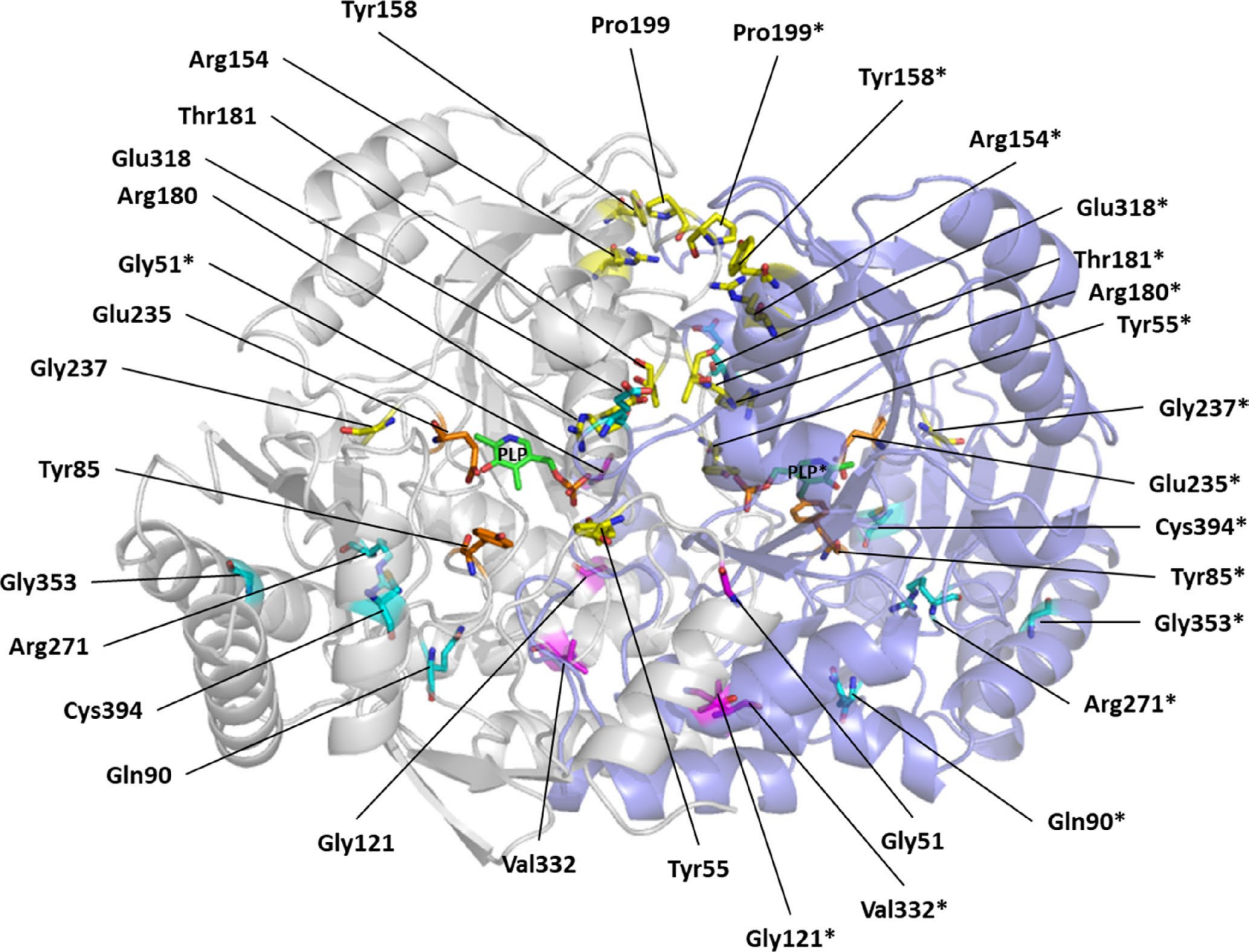
. Structural distribution of the main molecular effects of the characterized hOAT variants. Cartoon representation of the hOAT dimer structure. The monomers are colored in white and light blue, respectively. Residue subjected to mutation causing strong catalytic defect are colored in yellow or orange; residues whose mutations cause folding defect or PMP binding affinity reduction are colored in cyan and magenta, respectively. The Image was rendered by PyMol software (Schrödinger).

An important aspect of all these studies is not only to identify the molecular defects of each mutation causing disease(s), but also to unravel residues and/or regions that can be crucial for the folding of hOAT and/or for its catalytic properties. As illustrated in Figure 4, by mapping the position the of mutation sites exhibiting strong catalytic defect (yellow and orange residues), it is evident that the catalytic impairment is associated not only with the active site variants but also with mutations of residues located at the interface between the large domains of the monomers. For the majority of these residues, i.e., the yellow ones visible in the figure, the reduced catalytic efficiency is mostly ascribable to a strong reduction of the k_cat_ value or to an almost equal contribution of the k_cat_ value decrease and of the K_m_ value increase. The exceptions are residues Tyr85 and Glu235, highlighted in orange, whose mutation causes the reduction of the catalytic efficiency mainly because of an increased K_m_ value. This is not surprising, considering that both residues are involved in the substrates binding. It is noteworthy that almost all the yellow and orange residues highlighted in Figure 4 belong to flexible/not structured regions, such as loops 194–204, 172–184, 229–245. Therefore, these regions are definitely crucial for the acquisition of a catalytic competent conformation of the enzyme and a correlation between their alteration and the reduction of the turnover number of the enzyme is evident. Similarly, unstructured flexible regions crucial for the catalysis of other PLP dependent enzymes had already been identified [41, 42]; Indeed, in human dopa decarboxylase three flexible loops located at the monomers interface and/or the active site were identified as crucial structural regions for the apo‐ to ‐holoform transition and the catalytic activity of the enzyme [41].

On the other hand, mutations belonging to (i) N‐terminal or C‐terminal domains, (ii) the interface present between the N‐terminal and the large domain and (iii) other peripheral regions of the large domains (colored cyan and magenta in Figure 2) are reported to induce only modest effects on the catalysis [36, 37, 39]. These mutations (residues colored in cyan) are mainly responsible for folding defects, while in some other cases they reduced the PMP binding affinity (residues colored in magenta).

## 5 Conclusions

hOAT, a PLP‐dependent mitochondrial matrix enzyme, serves to form glutamate from Orn, and it is also involved in proline synthesis. Deficiency of hOAT causes gyrate atrophy, a rare inherited disease. The enzyme is also involved in other pathologies, such as hepatocellular carcinoma or lung cancer. The current state of the art in crystallographic and kinetic studies of hOAT, combined with the characterization of pathogenic and artificial variants of the enzyme, is presented and analyzed in this paper. Altogether, these studies allow us to identify the molecular defects of mutations associated with gyrate atrophy and to unravel residues and/or regions critical for catalytic and/or folding features of the enzyme. Nonetheless, research is still required to expand the molecular investigation to other hOAT variants and gain a deeper insight into the structural and functional properties of the enzyme. Both aspects are crucial in understanding the role of hOAT in the pathogenesis and can be helpful in the development of new therapeutic approaches.

## Author Contributions

**Fulvio Floriani:** data curation, visualization, writing – review and editing. **Carla Borri Voltattorni:** writing‐review and editing. **Riccardo Montioli:** conceptualization, writing – original draft, writing – review and editing.

## Conflicts of Interest

The authors declare no conflicts of interest.

## Data Availability

Data sharing not applicable to this article as no datasets were generated or analysed during the current study.
